# Assessment of primary health care facilities’ service readiness in Nigeria

**DOI:** 10.1186/s12913-017-2112-8

**Published:** 2017-03-01

**Authors:** Abayomi Samuel Oyekale

**Affiliations:** Department of Agricultural Economics and Extension, North-West University Mafikeng Campus, Mmabatho, 2735 South Africa

**Keywords:** Healthcare, Service readiness, Drug availability, Equipment availability, Nigeria

## Abstract

**Background:**

Effective delivery of healthcare services requires availability of adequate infrastructure, diagnostic medical equipment, drugs and well-trained medical personnel. In Nigeria, poor funding and mismanagement often characterize healthcare service delivery thereby affecting coverage and quality of healthcare services. Therefore, the state of service delivery in Nigeria’s health sector has come under some persistent criticisms. This paper analyzed service readiness of Primary Health Care (PHC) facilities in Nigeria with focus on availability of some essential drugs and medical equipment.

**Methods:**

Service Delivery Indicator (SDI) data for PHC in Nigeria were used. The data were collected from 2480 healthcare facilities from 12 states in the Nigeria’s 6 geopolitical zones between 2013 and 2014. Data were analyzed with descriptive statistics, Principal Component Analysis (PCA) and Ordinary Least Square regression.

**Results:**

Medical disposables such as hand gloves and male condoms were reported to be available in 77.18 and 44.03% of all the healthcare facilities respectively, while immunization services were provided by 86.57%. Functional stethoscopes were reported by 77.22% of the healthcare facilities, while only 68.10% had sphygmomanometers. In the combined healthcare facilities, availability of some basic drugs such as Azithromycin, Nifedipine, Dexamethasone and Misoprostol was low with 10.48, 25.20, 21.94 and 17.06%, respectively, while paracetamol and folic acid both had high availability with 74.31%. Regression results showed that indices of drug and medical equipment availability increased significantly (*p* < 0.05) among states in southern Nigeria and with presence of some power sources (electricity, generators, batteries and solar), but decreased among dispensaries/health posts. Travel time to headquarters and rural facilities significantly reduced indices of equipment availability (*p* < 0.05).

**Conclusion:**

It was concluded that for Nigeria to ensure better equity in access to healthcare facilities, which would facilitate achievement of some health-related sustainable development goals (SDGs), quality of services at its healthcare facilities should be improved. Given some differences between availability of basic medical equipment and their functionality, and lack of some basic drugs, proper inventory of medical services should be taken with effort put in place to increase funding and ensure proper management of healthcare resources.

## Background

The tenet of universal health coverage (UHC) in the post-2015 development agendas reemphasizes distributional equity and efficiency in healthcare service delivery, through provision of technical and financial supports to healthcare facilities at all levels of administering services [1, 2]. This is directly related to realization of several health-related targets in the Sustainable Development Goals (SDGs) [3, 4]. Although the world’s major health policy players—World Health Organization (WHO) and World Bank—have shown commitments towards deployment of requisite resources towards some of the set goals, several constraints on service readiness are often ignored at the national level of health planning [5]. This is often aggravated by existence of conflicting political ideologies on what is considered to be the best option in healthcare management [6, 7], budget constraints and persistence of some covariate and idiosyncratic economic shocks [8].

Although UHC is globally embraced as a prerequisite for significant economic development, the state of healthcare facilities in some developing countries contradicts their support to some global health development agendas. This is a serious matter given that the relevance of readily available and quality healthcare services for responding to emergencies in healthcare service demand cannot be over-emphasized. Assessing service readiness of healthcare facilities will broaden our understanding of their ability to adjust to some strategic changes [9–11].

In Nigeria, human capital development through provision of sound and efficient health delivery system is conceived as the bedrocks for economic growth and development [12, 13]. This ideology obviously guided economic planning and development agendas since the post colonial era. The primary proviso for reenergizing a national workforce that is able to drive development requisites in a manner that optimizes efficiency is perfectly encoded in systematically designed health service delivery system, among others.

Prescriptively amplified, health as necessary but insufficient inputs into national development processes energizes the population to tactically take crucial advantages of development opportunities [12]. Therefore, a country that is blessed with healthy people will optimize development initiatives through efficient utilization of technological innovations [14–16]. However, adequate infrastructure is required by any health care system to enhance delivery of services in an efficient, effective and timely manner. Such infrastructure defines the quality of services provided based on their relatively adjudged qualitative and quantitative characteristics [17, 18]. Beside the physical attractiveness of health infrastructure, their overall acceptability would be perceived from the notion of workability of the complementary technological and human resources, functionality of the road networks, water supply systems, electricity connectivity, e-readiness of the system and flexibility to adjust and be reintegrated with other future changes as more complex technological innovations unfold, among others [19, 20].

However, such requirements are presently absent at many healthcare facilities in many developing countries. Although lack of proper maintenance culture may be easily attributed to the observed decadence, inability to provide sufficient funds in order to replace old structures also contributes significantly. Therefore, since the past few decades, the state of service delivery in Nigeria’s health sector has come under some persistent criticisms [21–23]. It is imperative to reevaluate the preparedness of the healthcare facilities for service delivery in the spirit of working towards achieving health-related SDGs. This is very critical for Nigeria given its present poor performance in some health indicators. Specifically, WHO [24] stated that although Nigeria constituted less than 1% of the total world’s population, she accounts for about 19% of the global maternal deaths, with a maternal mortality ratio of 814 per 100,000 live births. In addition, although access to good quality obstetric care is critical for reducing maternal mortality, National Population Commission (NPC) [25] posited that in Nigeria, utilization of maternity care in 2013 was low and only about 36% of births occurred in health facilities with 38% being assisted by skilled personnel.

In Nigeria, differences exist between quality of healthcare services provided by private and public service providers, while some regional differences also exist. Specifically, Obi et al. [26] concluded that privately owned health facilities have better service readiness than public facilities. There exist some marked regional differences between socio-economic development in the northern and southern regions in Nigeria. Specifically, compared to southern Nigeria where Christianity is the predominant religion, residents in northern Nigeria are predominantly Muslims with their lifestyles resembling those of Arab states in North Africa and Middle East [27, 28]. It is important to note that judging by their religious inclination which was primarily introduced by foreign missionaries, residents in southern Nigerian are better educated with higher likelihood of embracing western lifestyles [27, 28]. The impacts of existing socio-political, ethnic, economic and religious diversities between northern and southern parts of Nigeria on health disparities manifest through differences in demand for healthcare services and households’ healthcare seeking behaviour [29, 30].

With respect to readiness of various healthcare facilities in providing efficient service delivery, Eboreime et al. [31] submitted that there are some gaps between access to healthcare facilities across Nigerian geopolitical zones. These disparities have been reported as the major supply-side factor affecting utilization of healthcare services. In some instances, Nigeria’s health care system has been found to operate below standards in terms of the availability of human resources and necessary infrastructure, equipment and medications. The result of a study by Eboreime et al. [31] indicated that although there was no significant association between geographical location and reported non-availability of immunization vaccines, the likelihood of accessing immunization within 5 km radius was higher for northern states than for those in southern states. However, available data show that in 2013, immunization coverage of zones in northern parts of Nigeria ranged between 14 and 44%, while in that for southern zones was between 70 and 81% [32].

Salako [33] noted that due to poor funding of the health sector and purchase of less important expensive drugs, healthcare facilities in many tropical African countries are unable to secure the needed drugs. It was emphasized that adoption of the WHO’s Essential Drug Programme (EDP) by Nigeria facilitated proper allocation of available funds on drugs that are required by many people. These drugs are also made available at affordable prices. Uzochukwu et al. [34] analyzed the effects of Bamako Initiatives (BI) on availability of essential drugs in Primary Health Care (PHC) facilities in South East Nigeria. This initiative was meant to facilitate operations of PHC in ensuring quality delivery of healthcare services in many African countries during the period of structural adjustments due to persistence of poor funding and associated inefficiency of many PHC delivery centers [35]. The study concluded that BI had positive impact on availability of essential drugs and efforts to address persistent problem of lack of essential drugs at non-BI healthcare facilities should be addressed. A study by Sambo et al. [36] assessed essential drugs’ availability and patients’ perceptions on the situation of drug availability at some PHC facilities in Tafa Local Government Area of North Central Nigeria. The results showed that none of the PHC implemented the Bamako Initiative while none operated the Drug Revolving Fund (DRF) system. It was concluded that resuscitating the Bamako Initiative would help some PHC in Nigeria to take availability of essential drugs very seriously in the course of service delivery.

## Methods

### Data and sampling methods

The health Service Delivery Indicator (SDI) data that were collected by the World Bank in Nigeria between 2013 and 2014 were used for this study. SDI survey aims to measure performance and quality of healthcare service delivery systems by collecting data on accuracy of diagnostics, compliance with basic clinical guidelines, caseloads, health staff absenteism, availability of drugs, medical equipment and infrastructure [37]. This study used the modules on availability of non-expired drugs and functioning basic medical equipment [38]. All the drugs and medical equipment for which availability was probed in the questionnaire were selected. These equipments and drugs are part of the requirements for minimum healthcare service delivery by PHC in Nigeria as recommended by the World Health Organization [39, 40].

The health facilities were selected using multi-stage cluster sampling by taking cognizance of the location (rural/urban) and the mode of operation (health posts/dispensaries/district hospitals). Detailed sampling procedures could be accessed from International Household Survey Network (IHSN) [41]. However, the data were collected with the goal of ensuring national representativeness This was ensured through consideration of geographic factor (rural/urban) and mode of operation. Multistage cluster sampling was used with the first level of stratification being the Local Government Areas (LGAs) (versus facilities) to ensure proper distribution of the samples across the geographic spread. The sampling frame was developed with due consideration fraction of public healthcare facilities, poverty rate and percentage of urbanization. The sampled healthcare facilities were classified into rural or urban and poor on non-poor [41]. The sampling involved selection of two states from each of the six geopolitical zones in Nigeria. Therefore, data were collected from 12 states, with a total of 2480 health facilities sampled. The frequency distribution of the selected healthcare facilities across the states and location (rural/urban) is provided in Table 1.

**Table 1:** Frequency distribution of sampled healthcare facilities in Nigeria, 2013/2014

States	Total
Anambra	199
Bauchi	212
Bayelsa	181
Cross River	205
Ekiti	208
Imo	230
Kaduna	215
Kebbi	209
Kogi	206
Niger	208
Osun	214
Taraba	193
Location	
Rural	1480
Urban	1000
Ownership type	
Public	2268
Private	199
Others	13
Mode of operation	
Dispensary	262
Health center	1667
District hospitals	275
Others	276
Total	2480

### Computation of service readiness indicators using equipment and drug availability

Consideration of healthcare facilities’ service readiness can be addressed from different perspectives. Although issues such as medical staff’s availability and competence are relevant, this paper focused on equipment and medications because the module on staff was not released for public use. In the data set, several variables were provided for each of these indicators, thereby warranting data aggregation in order to performs some further analyses. Principal Component Analysis (PCA) is able to reduce large number of variables into a composite index which would posses every feature found in the large data set. It is an excellent method to derive explicitly a variable of manageable magnitude and dimension from several variables which may actually possess different attributes [42]. The STATA software which was used for this study provides some post estimation commands, among which “predict” could be used to generate composite indices form the selected multiple dimensions.

### Ordinary least square (OLS) regression

OLS regression method was used to determine the factors explaining some composite indices that were generated from PCA. The analyses took cognizance of the problems of heteroscedasticity and multicollinearity. The former was addressed with Breusch-Pagan/Cook-Weisberg test. When this test shows statistical significance (*p* < 0.05), efforts should be made to address heteroscedasticity. In this study, robust standard errors were computed and used to evaluate statistical significance of the parameters. Multicollinearity was evaluated with variance inflation factor (VIF). This is a measure of the extent by which variance of the parameters had been inflated. The rule of thumb is that some cautions should be taken when VIF is up to 4, while serious model correction would be required if it is up to 10 [43].

### Analytical methods for health service delivery indicators

#### Determinants of drug and equipment availability

The estimated equations for the healthcare facilities are stated below:

1 $$ D R U{G}_i={\alpha}_1+{\beta}_k\;{\displaystyle \sum_{k=1}^{10}{X}_{i k}+{z}_i} $$

2 $$ EQUI{P}_i={\alpha}_2+{\varphi}_k\;{\displaystyle \sum_{k=1}^{10}{X}_{i k}+}{p}_i $$

Equation 1 will analyze the factors that would influence indices of drug availability, while Eq. 2 will determine the variables that would influence indices of equipment availability. From these two equations, *α*_1_, *β*_*k*_, *α*_2_, *φ*_*k*_, *α*_3_ and *γ*_*k*_ represent the parameters to be estimated. However, *Χ*_*ik*_ presents the vector of independent variables. These were coded a follows: rural health facility (yes =1, 0 otherwise), southern states (yes =1, 0 otherwise), time to travel of local headquarters (hrs), salaries paid by public sector (yes =1, 0 otherwise), running cost paid by public sector (yes =1, 0 otherwise), healthcare category (dispensaries/health center = 1, 0 otherwise), access to electricity (yes =1, 0 otherwise), access to generator (yes =1, 0 otherwise), access to batteries (yes =1, 0 otherwise) and access to solar panel (yes =1, 0 otherwise). In addition, z_i_, p_i_ and l_i_ are the stochastic error terms.

## Results

### Provision of immunization services and availability of medical disposables and vaccines

Table 2 shows the distribution of the healthcare facilities based on vaccination services and storage of vaccines. In the combined data, 86.6% of the health facilities provided vaccination services. However, only 13.6% of the combined healthcare facilities was able to store vaccines at their facilities. More specifically, the highest values were reported in Bauchi and Bayelsa states with 25.5 and 21.0%, respectively. Vaccines were stored in another healthcare facilities in 74.1% of the combined data. This is understandable given the fact that only 28.6% of the health facilities in the combined data indicated availability of refrigerators.

**Table 2:** Immunization services and storage of vaccines by healthcare facilities in Nigeria, 2013/2014

	Anambra	Bauchi	Bayelsa	Cross River	Ekiti	Imo	Kaduna	Kebbi	Kogi	Niger	Osun	Taraba	Total
Immunization services provided	94.0	88.2	82.3	91.7	89.4	81.7	82.3	94.7	75.2	91.8	83.2	84.5	86.6
Vaccines stored at the facility	10.6	25.5	21.0	15.6	10.6	10.0	13.5	15.3	7.8	14.9	14.5	3.6	13.6
Vaccines stored at another facility	83.9	63.2	63.0	76.1	79.3	71.7	69.3	79.9	67.5	81.3	71.5	82.4	74.1
Vaccine carrier(s)	93.5	78.3	76.8	85.4	78.9	78.7	75.8	76.1	69.4	77.4	77.1	65.8	77.8
Refrigerator available	34.7	29.3	44.8	24.4	49.0	28.7	27.4	23.9	23.3	14.9	36.9	6.7	28.6

Table 3 shows the percentage distribution of availability of some medical disposables and vaccines at the selected health facilities. It reveals that although disposable gloves were available in 77.2% of the combined healthcare facilities, Kebbi state reported the lowest value of 37.3%. Male condoms were available in 44.0% of the combined healthcare facilities, although Kebbi and Niger states had the values of 19.6 and 27.8%, respectively. Majority of the healthcare facilities reported to be rendering immunization services. Specifically, Anambra, Kebbi and Niger states respectively reported highest values with 94.0, 94.7 and 91.8%, respectively. Majority of the healthcare facilities did not have vaccines. In the combined data, 10.4% of the healthcare facilities indicated availability of measles, BCG and Hepatitis B vaccines respectively, while 11.1 and 11.0% respectively had polio and DTP-Hib + HepB (pentavalent) vaccines. Table 3 shows that only 28.6% of the healthcare facilities in the combined data indicated availability of refrigerators.

**Table 3:** Percentage distribution of availability of medical disposables, vaccines and refrigerators at the healthcare facilities in Nigeria, 2013/2014

	Anambra	Bauchi	Bayelsa	Cross River	Ekiti	Imo	Kaduna	Kebbi	Kogi	Niger	Osun	Taraba	Total
Disposable gloves	90.0	72.6	89.5	85.4	83.2	90.9	85.1	37.3	72.3	86.1	61.7	73.1	77.2
Male condoms	56.3	52.4	41.4	53.7	38.5	36.5	62.8	19.6	40.8	27.9	50.5	48.7	44.0
Measles vaccine & diluents	6.5	15.1	20.4	8.3	8.2	9.6	12.1	11.0	5.8	11.1	13.6	3.1	10.4
Oral Polio Vaccine (OPV)	7.5	20.8	19.3	10.7	8.7	8.3	12.1	11.5	6.8	13.5	13.1	1.6	11.1
Diphteria + pertussis + tetanus vaccine (DPT/Trivalent)	1.0	10.9	1.7	5.9	1.0	0.4	2.3	4.3	0.5	4.3	0.0	2.1	2.9
DTP-Hib + HepB (pentavalent) vaccine	8.5	19.3	19.3	10.2	8.7	9.6	10.7	10.5	6.8	13.5	13.6	1.6	11.0
Pneumococcal (PCV 10) vaccine	2.0	3.3	2.2	2.0	0.5	2.2	3.3	4.8	0.0	0.5	0.5	0.5	1.8
BCG vaccine & diluents	7.0	20.3	18.8	10.7	8.2	8.7	8.8	9.1	6.3	12.5	11.7	2.6	10.4
Hepatitis B vaccine	7.5	19.3	16.0	10.7	8.2	9.6	10.7	11.5	6.3	12.5	10.8	2.1	10.4
Disposable syringes with disposable needles	94.0	74.5	79.6	84.9	80.8	76.5	75.8	72.7	70.4	87.0	77.1	68.9	78.5

### Availability of functioning medical equipment

Table 4 shows the availability of some medical equipment by the selected healthcare facilities. It reveals that not all the equipment that were present at the healthcare facilities were in good working condition. Adult weighing scale was present in 94.5%, of the health facilities in Anambra state, although only 85.4% was functioning. The states with lowest functioning adult weighing scales were Taraba (51.8%), Kebbi (52.2%), Niger (58.7%) and Bauchi (59.4%). Availability of functioning infant weighing scale was reported by 69.1, 65.9 and 65.8% of the respondents from Bayelsa, Ekiti and Anambra states. Similarly, child weighing scales were least found in health facilities in Niger, Kebbi and Taraba states with 30.8, 32.5 and 33.7%, respectively. However, it was only in Kaduna state that more than half of the child’s weighing scales were functioning.

**Table 4:** Percentage Distribution of availability and functionality of medical equipment in healthcare facilities in Nigeria, 2013/2014

	Anambra	Bauchi	Bayelsa	Cross River	Ekiti	Imo	Kaduna	Kebbi	Kogi	Niger	Osun	Taraba	Total
Adult weighting scale	94.5	64.2	85.1	85.9	96.6	83.0	88.8	57.4	77.2	69.2	90.2	56.0	79.1
Adult weighting scale functional	85.4	59.4	80.7	76.6	90.9	72.2	81.4	52.2	66.5	58.7	76.2	51.8	71.0
Thermometer present	91.0	54.7	87.3	88.3	89.4	86.5	84.2	58.9	73.8	78.4	78.0	61.1	77.6
Thermometer functional	87.9	51.4	85.1	82.9	86.5	83.0	78.6	52.6	69.9	72.6	73.8	60.1	73.7
Chifgraphld weighting scale present	51.3	46.2	44.2	57.6	45.7	38.7	60.9	49.8	37.4	49.5	46.3	42.0	47.5
Child weighting scale functional	45.7	40.1	39.2	48.3	39.9	34.4	54.9	41.6	31.1	40.4	40.7	38.9	41.3
Stethoscope present	90.5	72.6	86.2	86.3	95.7	89.1	91.6	64.1	72.3	88.9	86.5	69.4	82.9
Stethoscope functional	81.9	68.4	82.9	81.0	89.4	84.4	87.0	56.5	65.5	86.1	77.1	65.8	77.2
Infant weighting scale present	70.9	53.3	77.9	60.0	71.6	66.5	58.6	40.2	44.7	34.1	59.4	37.3	56.1
Infant weighting scale functional	65.8	49.5	69.1	50.2	65.9	60.0	50.2	32.5	37.9	30.8	55.6	33.7	50.0
Sphygmomanometer present	87.4	66.5	87.3	84.4	91.8	87.8	86.5	55.5	64.6	84.1	81.8	66.8	78.8
Sphygmomanometer functional	75.4	51.4	81.2	70.7	79.3	80.0	74.0	47.4	55.8	75.5	65.4	61.7	68.1
Autoclave present	17.1	18.4	41.4	18.5	31.3	16.1	26.5	13.4	21.8	9.6	28.5	18.1	21.5
Autoclave functional	9.6	14.6	24.3	11.2	21.6	10.4	20.9	9.6	14.1	8.2	22.4	13.0	14.9
Electric boiler/steamer present	3.5	12.7	27.1	14.6	19.7	9.6	18.6	12.4	15.1	7.7	22.4	7.8	14.2
Electric boiler/steamer functional	3.5	13.2	27.1	14.6	17.3	9.6	18.6	12.4	15.1	8.2	22.4	7.8	14.1
Electric dry heat sterilizer present	9.6	10.4	22.1	9.3	15.4	8.3	12.6	13.9	10.7	9.6	13.1	8.8	11.9
Electric dry heat sterilizer functional	6.0	7.1	13.8	5.9	7.2	5.2	9.3	8.6	6.8	7.2	11.7	5.7	7.8

Thermometers were found in 91.00% of the healthcare facilities in Anambra state, while only 58.9% of those from Kebbi had it. Functioning thermometers were least reported in Kebbi and Taraba states with 52.6 and 60.1%, respectively. Functioning stethoscopes were least found in health facilities in Kebbi state and Kogi state with 56.5 and 65.5% respectively. The states with highest percentages having functioning stethoscopes were Ekiti, Kaduna and Niger with 89.4, 87.00 and 86.1%, respectively. Sphygmomanometers were most functioning in health facilities in Bayelsa, Imo and Ekiti states with 81.2, 80.0 and 79.3%, respectively. The states that had the least percentages of functioning sphygmomanometers were Kebbi and Bauchi states with 47.4 and 51.4%, respectively.

It should be noted that out of the 41.4% of the healthcare facilities that reported to have autoclaves in Bayelsa state, only 24.3% indicated that they were in good working condition. Availability of autoclaves was very low in Niger state with 9.6%, while only 8.2% reported that they were functioning. Other states with low percentages reporting functioning autoclaves were Anambra and Kebbi states with 9.6%.

Majority of the healthcare facilities reported absence of functioning electric boilers. Specifically, Bayelsa, Osun and Kaduna states had the highest availability with 27.1, 22.4 and 18.6%, respectively. Those states with the lowest availability of functioning electric boilers were Anambra, Taraba and Niger states with 3.5, 7.8 and 8.2%, respectively. Similar results were obtained for availability of functioning electric dry heat sterilizer with Bayelsa and Osun states having highest availability with 13.8 and 11.7%, respectivbely. Moreover, Imo, Taraba and Cross River states reported the lowest availability with 5.2, 5.7 and 5.9%, respectively.

Table 5 presents the descriptive statistics of the computed indices for equipment availability. It reveals that northern states were generally with the lowest average equipment availability indices. Specifically, Bayelsa and Ekiti states had the highest average values with 0.6 and 0.7, respectively, while Kebbi and Taraba had the lowest average values with −0.9 and −0.6. The facilities in urban centers had higher average equipment indices with 0.5, as compared with −0.4 for rural healthcare facilities. Privately owned healthcare facilities also had higher average equipment index with 0.9, which can be compared with −0.1 for public healthcare facilities. District hospital had highest average equipment index with 1.8.

**Table 5:** Descriptive statistics of equipment availability indices in healthcare facilities in Nigeria, 2013/2014

	Frequency	Mean	Std Dev
*State*			
Anambra	199	0.3	1.3
Bauchi	212	−0.5	2.0
Bayelsa	181	0.7	1.8
Cross River	205	0.2	1.5
Ekiti	208	0.6	1.3
Imo	230	0.2	1.5
Kaduna	215	0.5	1.8
Kebbi	209	−0.9	2.0
Kogi	206	−0.4	1.9
Niger	208	−0.2	1.6
Osun	214	0.2	1.9
Taraba	193	−0.6	2.1
*Location of facility*			
Rural	1480	−0.4	1.8
Urban	1000	0.5	1.7
*Ownership*			
Public	2268	−0.1	1.8
Private	199	0.9	1.9
*Mode of Operation*			
Dispensary	262	−2.0	1.4
Health Centre	1667	−0.1	1.6
District Hospital	275	1.8	1.5
Other	102	0.9	1.6

### Availability of basic drugs

Table 6 shows the distribution of the health facilities based on availability of non-expired drugs. It reveals that only 8.7, 8.1 and 13.6% of the healthcare facilities in Ekiti, Kebbi and Osun states respectively had non-expired Ceftriaxone. Healthcare facilities in Bayelsa and Kaduna states recorded the highest availability of non-expired Ceftriaxone with 30.4 and 33.0%, respectively. Availability of non-expired Diazepan was highest in healthcare facilities from Anambra and Bayelsa states with 56.3 and 53.6%, respectively. Non-expired Oxytocin was available in 18.2 and 27.9% of the healthcare facilities from Kebbi and Osun states, respectively. These are the lowest percentages among the selected states.

**Table 6:** Percentage distribution of availability of non-expired drugs in selected healthcare facilities in Nigeria, 2013/2014

Drug Name	Anambra	Bauchi	Bayelsa	Cross River	Ekiti	Imo	Kaduna	Kebbi	Kogi	Niger	Osun	Taraba	Total
Antibiotics													
Ceftriaxone	24.6	15.6	30.4	14.2	8.7	20.9	33.0	8.1	23.3	13.0	13.6	21.2	18.8
Ampicillin	35.2	14.6	29.3	22.9	16.4	28.3	37.2	9.6	25.2	20.7	14.5	27.5	23.4
Gentamicin	72.9	23.6	60.8	52.2	49.5	76.1	58.6	16.3	62.6	68.8	42.5	59.6	53.6
Metronidazole	70.9	38.7	68.0	54.6	70.7	71.7	74.0	23.9	62.6	61.1	57.0	66.8	59.9
Azithromycin	11.6	3.3	22.1	13.2	5.3	11.3	20.5	7.7	9.7	9.1	5.1	8.3	10.5
Benzanthine benzyl penicillin	39.2	16.5	33.2	42.0	20.7	52.2	43.3	11.0	33.0	53.4	29.0	38.3	34.4
Chloramphenicol	41.2	29.7	27.6	12.2	24.0	30.9	43.3	16.3	31.1	38.5	29.0	27.5	29.3
Amoxicilline for children (125 mg)	50.3	43.4	58.0	37.1	48.1	45.2	62.8	14.8	46.6	46.6	62.2	48.7	46.9
Benzylpenicillin for children	44.7	21.2	33.7	43.4	25.5	51.3	47.4	12.4	36.4	54.8	29.4	44.6	37.1
Anesthetics													
Diazepam	56.3	21.7	53.6	33.7	40.4	41.3	43.3	10.5	31.6	31.7	39.3	37.3	36.5
Oxytocin													
Oxytocin (Syntocinon)	56.3	33.0	55.8	42.9	27.9	58.7	51.6	18.2	38.8	46.2	32.2	41.5	41.9
Ergometrine	53.3	34.4	58.6	56.6	54.3	64.8	46.5	19.6	50.5	50.0	44.9	56.5	49.1
Gastro-Intestinal													
Magnesium Sulphate	10.6	15.1	27.6	10.7	14.9	17.0	26.1	17.2	14.1	17.3	7.9	9.8	15.7
Oral rehydration solution (ORS)	73.4	47.6	70.2	36.6	66.4	60.0	60.9	27.3	48.1	60.1	37.4	53.9	53.3
Zinc for children	56.8	29.3	34.3	13.2	3.9	41.7	24.7	11.0	17.0	9.1	18.7	9.3	22.4
Misoprostol (Mifepristone)	19.1	15.1	31.5	7.3	16.4	17.0	40.0	10.1	10.7	16.4	12.6	9.3	17.1
Hypertension Drug													
Nifedipine	34.2	24.1	49.2	15.1	20.7	30.9	43.7	10.1	24.8	10.6	16.8	24.9	25.2
Anti-malaria													
Artemisinin combination therapy for children	84.4	63.7	66.3	58.5	70.2	72.2	52.1	15.3	67.5	54.3	70.1	43.0	59.8
Anti anaemia													
Ferrous salt	74.9	48.1	64.6	68.8	73.6	75.2	74.9	22.5	71.8	65.4	63.6	72.0	64.6
Folic acid	81.9	59.4	80.1	76.6	79.3	85.2	80.5	30.1	81.1	73.1	86.5	78.2	74.3
Mineral supplements													
Calcium Gluconate	26.1	6.6	22.1	11.2	7.2	22.2	15.8	5.7	16.0	5.8	7.9	6.2	12.7
Sodium Chloride (Saline Solution/NaCl)	42.2	24.1	58.0	29.3	32.2	45.2	47.9	12.9	41.3	35.1	32.2	37.3	36.3
Vitamin A for children	76.4	45.3	65.2	57.6	58.2	62.2	36.7	14.4	39.3	32.7	33.6	35.2	46.2
Anti-Allergies													
Betamethasone or Dexamethasone	40.7	18.9	38.1	17.1	11.5	20.9	29.8	8.1	29.1	16.8	12.2	23.3	21.9
Contraceptive													
Medroxyprogesterone acetate	45.2	44.8	26.5	36.6	16.8	19.1	47.9	12.9	21.4	27.4	27.6	30.6	29.7
Pain and Palliative Care													
Paracetamol	87.4	48.1	78.5	72.7	83.7	91.3	82.3	34.5	85.4	85.6	60.8	82.4	74.3
Anthelminthics													
Albendazole for children	65.3	58.0	62.4	70.7	54.8	50.4	63.3	15.3	54.4	47.6	29.4	63.7	52.7

The states with highest availability of non-expired Calcium Gluconate were Anambra and Imo with 26.1 and 22.2%, respectively. However, this medicine was least found in Kebbi, Niger and Bauchi states with 5.7, 5.8 and 6.6%, respectively. Non-expired Magnesium Sulphate was available mostly in Bayelsa state with 27.6%, while Osun state had least availability with 7.9%. Non-expired Sodium Chloride (Saline Solution) was available in 36.3% of all the health facilities that were selected, while non-expired Misoprostol (Mifepristone) was reported in 17.06%. In all the healthcare facilities, the non-expired medicines that were readily availability included Ferrous Salt, Folic Acid and Paracetamol.

The indices of drug availability indices are presented in Table 7. The results show that among the states, Anambra had the highest average index of 1.3, while Kebbi had the lowest value (−2.6). Similarly, private and urban health facilities had higher average drug availability indices with 1.6 and 0.4, respectively. Also, district hospitals had the highest drug availability index with 2.9.

**Table 7:** Descriptive statistics of drug availability indices in healthcare facilities in Nigeria, 2013/2014

	Frequency	Mean	Std Dev
*State*			
Anambra	199	1.3	2.9
Bauchi	212	−0.9	3.1
Bayelsa	181	1.1	3.2
Cross River	205	−0.2	2.6
Ekiti	208	−0.1	2.6
Imo	230	0.9	2.8
Kaduna	215	1.1	3.2
Kebbi	209	−2.6	3.3
Kogi	206	0.0	3.0
Niger	208	−0.1	2.8
Osun	214	−0.8	2.6
Taraba	193	0.4	2.8
*Location*			
Rural	1480	−0.3	3.1
Urban	1000	0.4	3.1
*Ownership*			
Public	2268	−0.1	3.0
Private	199	1.6	3.4
*Mode of operation*			
Dispensary	262	−2.8	2.5
Health Centre	1667	−0.1	2.8
District Hospital	275	2.9	2.5
Other	102	0.9	3.3

#### Determinants of equipment availability index

Table 8 shows the results of Ordinary Least Square regression of the factors explaining functioning equipment indices. From the F-statistics, the results show that the model was statistically significant (*p* < 0.01). The adjusted coefficient of determination implies that the model explained 32.7% of the variations in the values of equipment availability indices. The parameter of southern states had positive sign and it was statistically significant (*p* < 0.01). This implies that compared to their counterparts from northern Nigeria, medical equipment availability increased by 0.2307 for those medical facilities from states in southern part of Nigeria.

**Table 8:** Determinants of healthcare facilities’ functioning equipment indices in Nigeria, 2013/2014

	Equipment availability index		
	Coefficients	t stat	VIF
Southern states	0.2307^a^	3.56	1.17
Rural facility	−0.1623^b^	−2.4	1.23
Traveling time to headquarters	−0.2016^a^	−4.19	1.17
Public paid for medical supplies	0.0748	0.63	1.79
Public paid for running costs	−0.0177	−0.19	1.73
Dispensaries/health center	−0.8828^a^	−10.86	1.28
Electricity	1.0805^a^	14.5	1.56
Generator as second power source	0.6941^a^	8.14	1.53
Batteries as second power source	0.7143^b^	2.00	1.03
Solar panel as second power	0.6601^a^	3.18	1.06
Constant	0.3014^c^	1.84	
Number of observation	2480		
F - test	119.75^a^		
Adj R-squared	0.3266		
Mean VIF			1.33

^a^significant at 1%

^b^significant at 5%

^c^significant at 10%

However, medical equipment indices reduced significantly (*p* < 0.05) by 0.1623 for those healthcare facilities in rural areas, when compared to their urban counterparts. This was also expected because health facilities in rural Nigeria had been judged to be deficient in requisite medical equipment. The results also showed that if the number of hours of traveling to local headquarters from the health facilities increases by one unit, equipment index reduces by 0.2016. The health facilities that were classified as dispensaries/health center had their equipment index being significantly lower by 0.8828 when compared with the other class of healthcare facilities. In addition, healthcare facilities with access to electricity, generator and solar panel had significantly higher equipment indices.

#### Determinants of drugs availability indices

The results in Table 9 show the factors explaining drug availability within the selected health facilities. A comprehensive list of drugs which the questionnaire probed into their availability in non-expiry form is presented in Table 6. The list was used to generate drug availability indices using the Principal Component Analysis (PCA). Subjecting the generated indices to Ordinary Least Square regression presents the results in Table 9. The results show that 19% of the variations in the values of drug availability indices had been explained by the included explanatory variables. The F-statistics also showed statistical significance (*p* < 0.01). This implies that the hypothesis that all included variables were jointly statistically insignificant should be rejected.

**Table 9:** Determinants of healthcare facilities’ drug availability indices in Nigeria, 2013/2014

	Drug availability index		
	Coefficients	t stat	VIF
Southern states	0.2615^b^	2.17	1.17
Rural facility	0.1796	1.42	1.23
Traveling time to headquarters	−0.0291	−0.33	1.17
Public paid for medical supplies	−0.2071	−0.94	1.79
Public paid for running costs	−0.2283	−1.29	1.73
Dispensaries/health center	−1.3391^a^	−8.85	1.28
Electricity	1.1581^a^	8.35	1.56
Generator as second power source	1.3099^a^	8.25	1.53
Batteries as second power source	1.8421^a^	2.77	1.03
Solar panel as second power	0.8676^b^	2.25	1.06
Constant	0.2359	0.78	
Number of observation	2480		
F-test	59.16^a^		
Adj R-squared	0.1900		
Mean VIF			1.33

^a^significant at 1%

^b^significant at 5%

The results show that the parameter of southern states shows statistical significance (*p* < 0.05). This implies that indices of drug availability increases by 0.2615 for those states from southern parts of Nigeria, when compared with their counterparts from northern Nigeria. Furthermore, the parameter of dispensaries/health centers shows statistical significance (*p* < 0.01). This shows that drug indices reduced by 1.3391 among those health facilities that were classified as dispensaries/health centers when compared with the other types of health facilities. The results further show that drug indices increased significantly among health facilities with access to electricity, generators, solar panel and batteries.

## Discussions

Low possession of some essential drugs and medical disposables in many of the selected healthcare facilities is worrisome. Specifically, lack of hand gloves in some healthcare facilities raises some serious concerns. WHO [44] noted that different forms of disposable gloves are used during healthcare service delivery. These include the gloves wore during medical examination such as surgical gloves, sterile or non-sterile gloves, and chemotherapy gloves. When gloves are not available at some certain health centers, this can be very risky due to higher likelihood of transmitting germs from patients to doctors and nurses. Healthcare service providers (nurses and doctors) can then in turn transmit such pathogens to other people including their patients. WHO [45] however noted that use of glove by healthcare service givers should not replace the essentiality of hand washing hygiene. It was recommended that washing of hands should be done before and after wearing hand gloves.

Male condoms were not readily available in many of the selected healthcare facilities. Condom as a viable means of protective sexual intercourse is able to safeguard unwanted pregnancy and transmission of sexually transmitted infections (STIs). It has been widely acknowledged that beside abstinence which guarantees perfect protection from HIV and other STIs, condom use promises to safeguard contraction of infection through sexual activities [46]. In rural Nigeria, low usage of condom had been reported, most importantly among those who were single. Also, poor knowledge of reproductive issues are directly linked to unwanted pregnancy in Nigeria. In a study by Oyediran et al. [47], about 43.9% of adolescents who were attending schools in Ibadan lacked knowledge on likelihood of getting pregnant from the first coitus. Teenage sexual behaviour often adds significantly to the burden of STI, HIV transmission, abandoned children and socioeconomic deprivations in Nigeria [47–49].

Immunization services were largely rendered by the healthcare facilities. This goes in line with expectation that as the closest form of health service to the masses, PHC takes immunization very seriously as a way giving some form of preventive healthcare services. The 1978 Declaration of Alma-Ata clearly described PHC as “essential health care based on practical, scientifically sound and socially acceptable methods and technology made universally accessible to individuals and families in the community through their full participation and at a cost that the community and country can afford to maintain at every stage of their development in the spirit of self-reliance and self determination” [50]. In addition, as the closest health service delivery centers to the people, PHC is expected to be a functioning system that engages the services of different health professionals for the promotion of health services (including preventive, treatment, health supports and rehabilitation) for ensuring physical, emotional and mental well-being of users. With the Federal Ministry of Health spearheading health-related policies in Nigeria, the 1987’s National Health Policy seeks to provide comprehensive health care delivery system, which is driven by primary health care [51].

However, majority of the healthcare facilities lacked some means of storing vaccines. It will therefore be very difficult to guarantee availability of immunization services at any point in time. This can also be linked to non-availability of regular sources of power supply, which are required for proper usage of refrigerators. It should be noted that although many healthcare facilities indicated availability of other sources of power such as generator, batteries and solar, the intensity of usage was not probed into in the use. Therefore, high running cost of generator may prevent its regular usage. However, it could be concluded that the National Programme on Immunization (NPI) emphasized the different categories of immunization that are required by children and the healthcare facilities were making some efforts at providing them [52].

The quality of services rendered at healthcare facilities can be properly gauged from availability of medical equipment and drugs. In this study, cognizance was taken of the minimum standard required of PHC in Nigeria [39]. Based on this minimum standard, some basic equipment and medications are expected to be found in a PHC facility. This is essential in order to facilitate delivery of timely and efficient services to healthcare users. One major observation was that some medical equipment at the sampled healthcare facilities were no longer functioning. This may be obviously linked to lack of adequate funding or inability of the healthcare facilities to prioritize the need for putting some medical equipment in functional state. Omoluabi [53] however emphasized that among several other constraints, lack of medical equipment is a major problem affecting Nigerian health sector.

Medical equipment of high availability and functionality included adult weighing scale, thermometer and stethoscopes. The implication was that about some healthcare facilities would not be able to take blood pressure of patients, while majority would not be able to measure the weight of children. Electric dry heat sterilizers, autoclaves and electric boilers were generally lacking in the selected PHC facilities. Similarly, the worse results for equipment indices were obtained for states in northern Nigeria except Kaduna. Bauchi and Kebbi states are considered as hot spots for some specific interventions given their low possession of all the medical equipment. Poor availability of medical equipment in Nigerian PHC facilities had been previously reported [51, 54].

Also, availability of non expired drugs was low for some of the listed drugs. The implication is that patients in need of those essential drugs would have to source for them elsewhere. It also implies that ability to respond to emergencies in relation to some commen illnesses would be limited as a result of non-availability of drugs. Specifically, paracetamol and folic acid are the major drugs that were present at the healthcare facilities. But these are common drugs for which no expertise is needed before they could be sold to people in Nigeria.. It is also worrisome that anti-malaria drug for children was not readily available at some healthcare facilities despite the fact that malaria is a major health problem among Nigerian children,

Some of these results obviously allude to assertion of National HIV/AIDS Division et al. [55] that better healthcare facilities are accessible in southern Nigeria and that qualified medical staff are not easily attracted to northern part of Nigeria. Omoluabi [53] similarly noted that healthcare in urban and southern Nigeria are better equipped with medical personnel than most of the ones in northern Nigeria. It was highlighted that working condition, availability of medical equipment and some intangible benefits would among others influence ability to retain a qualified medical staff in Nigeria. Similarly, insurgencies in northern Nigeria and high poverty rate could also affect ability to retain qualified medical staff. Ojora-Saraki [56] noted that the growing insecurity in northern Nigeria is a major problem to healthcare service delivery. Poor availability of drugs in the health facilities is a confirmation to several studies like Sambo et al. [36], Ohuabunwa [54], and Ehiri et al. [57].

## Conclusion

The mandate of UHC is very important for fast tracking human development in Nigeria. This is so due to high level of productivity and welfare losses that are associated with morbidity and disease burdens in the country. In the light of pursuing the recently launched SDGs, evaluation of healthcare service quality becomes imperative given that over and above the physical buildings, services rendered at healthcare facilities are the direct inputs required to influence recovery of sick people. When these are deficient, a situation of skeptic development process ensues. This study unfolded the state of medical equipment and availability of drugs in Nigeria healthcare facilities. The findings have shown some variances between availability of basic medical equipment and their functionality. It was also noted that basic drugs are not readily available at the selected health facilities, thereby compromising service readiness of the healthcare facilities. This presupposes that majority of the healthcare facilities could not meet the minimum standard for PHC service delivery. It is therefore recommended that efforts to reevaluate and take inventory of services rendered at PHC in order to inform policies that would enhance their service quality and readiness should be channeled. Government should also reevaluate state of services delivered in rural healthcare facilities with a view of reequipping them with necessary drugs and medical equipment. Given that essential drugs were poorly available, there is the need for ensuring that leakages in drug acquisition and usage at healthcare facilities are removed. It was not so clear whether the drugs were diverted for personal uses or they were never procured. A critical evaluation of expenses at PHC would unfold what may have transpired. Therefore, proper auditing of PHC is recommended while the need to ensure adequate provision of power cannot be compromised.

## Abbreviations

AIDS: Acquired Immune Deficiency Syndrome
BCG: Bacilli Calmette Guerin
BI: Bamako Initiatives
DPT: Diphtheria, Pertusis, Tetanus
DRF: Drug Revolving Fund
FGD: Focus Group Discussions
HIV: Human Immunodeficiency Virus
IHSN: International Household Survey Network
LGA: Local Government Authorities
MDG: Millennium Development Goal
NPC: National Population Commission
NPHCDA: National Primary Health Care Development Agency
NPI: National Programme on Immunization
OLS: Ordinary Least Square regression
OPV: Oral Polio Vaccine
PCA: Principal Component Analysis
PHC: Primary Health Care
SDG: Sustainable Development Goal
STI: Sexually Transmitted Infection
UHC: Universal Health Coverage
VIF: Variance Inflation Factor
WHO: World Health Organization
YCHC: Yakawada Comprehensive Health Centre
